# Tissue Barriers to Arbovirus Infection in Mosquitoes

**DOI:** 10.3390/v7072795

**Published:** 2015-07-08

**Authors:** Alexander W.E. Franz, Asher M. Kantor, A. Lorena Passarelli, Rollie J. Clem

**Affiliations:** 1 Department of Veterinary Pathobiology, University of Missouri, Columbia, MO 65211, USA; E-Mails: franza@missouri.edu (A.W.E.F.); amkt33@mail.missouri.edu (A.M.K.); 2 Division of Biology, Kansas State University, Manhattan, KS 66506, USA; E-Mail: lpassar@ksu.edu

**Keywords:** arbovirus, mosquito, midgut, dissemination, salivary gland, tissue barrier, basal lamina, midgut infection barrier, midgut escape barrier

## Abstract

Arthropod-borne viruses (arboviruses) circulate in nature between arthropod vectors and vertebrate hosts. Arboviruses often cause devastating diseases in vertebrate hosts, but they typically do not cause significant pathology in their arthropod vectors. Following oral acquisition of a viremic bloodmeal from a vertebrate host, the arbovirus disease cycle requires replication in the cellular environment of the arthropod vector. Once the vector has become systemically and persistently infected, the vector is able to transmit the virus to an uninfected vertebrate host. In order to systemically infect the vector, the virus must cope with innate immune responses and overcome several tissue barriers associated with the midgut and the salivary glands. In this review we describe, in detail, the typical arbovirus infection route in competent mosquito vectors. Based on what is known from the literature, we explain the nature of the tissue barriers that arboviruses are confronted with in a mosquito vector and how arboviruses might surmount these barriers. We also point out controversial findings to highlight particular areas that are not well understood and require further research efforts.

## 1. Introduction

Most arbovirus vectors are hematophagous insects belonging to the order *Diptera*, such as mosquitoes, sandflies, black flies, and biting midges [1]. Further, numerous viruses of the families *Flaviviridae* and *Bunyaviridae* are transmitted by hard ticks (class: *Arachnida*). It is important to note that most species of insects and ticks do not serve as vectors for arboviruses. Even within insect or tick species, there is often considerable variation in vector competence among populations. Thus, there is considerable specificity in the vector-arbovirus relationship, and some of this specificity comes from the ability of a particular arbovirus to overcome tissue barriers in the vector to establish a persistent infection. The majority of medically-important arboviruses causing severe morbidity and mortality in humans around the world belong to the families *Flaviviridae*, *Togaviridae*, and *Bunyaviridae* and are transmitted by mosquitoes [2]. With the exception of O’nyong-nyong virus (ONNV), which is transmitted by anopheline mosquitoes, all other known mosquito-borne viruses are transmitted by culicine mosquitoes, mainly in the genera *Aedes* and *Culex*. The majority of *Culex*-transmitted arboviruses, belonging to the *Flaviviridae* and *Togaviridae*, have avian reservoirs and cause encephalitis-like illnesses in human hosts [3]. Most arboviruses of the families *Flaviviridae* and *Togaviridae* transmitted by *Aedes* *spp*. have either primate reservoirs or circulate between humans and mosquitoes, without an additional animal reservoir in so-called sylvatic or urban cycles, respectively. Typically, *Aedes*-transmitted arboviruses are associated with hemorrhagic disease manifestations in their human hosts.

Arbovirus disease mitigation principally relies on vector control strategies including residual insecticide spraying, removal of potential mosquito oviposition sites near human habitats, and the use of insecticide-treated bed nets and/or window curtains or screens [4]. However, due to increasing transcontinental trade and human movement, the prevalence of vector-borne diseases have dramatically increased in disease-endemic countries and have spread to new regions of the world [5,6,7,8]. As a result, public health authorities in tropical countries are often overwhelmed with increasing disease burdens. The situation is becoming more complicated as resistance to insecticides such as pyrethroids and organo-phosphates is spreading among mosquito populations [9,10]. Further, licensed human vaccines are available for the control of only a handful of arboviruses [7,11]. Thus, there is a need to increase the “tool box” for arbovirus control by supplementing existing control strategies with promising novel strategies that focus on interrupting the viral disease cycle in the vector. These novel arbovirus control strategies include genetic pest management techniques in which molecular genetic tools are employed to either reduce mosquito populations in target areas or to modulate their vector competence for arboviruses (population replacement) [12,13,14]. Manipulation of arbovirus vector competence, however, requires a thorough understanding of the factors that determine the vector competence of a mosquito for a virus.

Here, we provide a detailed description of what is known about how arboviruses establish systemic and persistent infections in competent mosquito vectors. We describe the basic anatomy of tissues relevant to arbovirus infection, such as the alimentary canal and salivary glands, and the barriers to infection within these tissues. The complex natures of midgut and salivary gland infection/escape barriers are described based on findings from the literature. We then propose mechanisms by which arboviruses might overcome these barriers. A novel signaling pathway involving fibroblast growth factor (FGF), matrix-metalloproteinases (MMP) and caspases has been described for the dissemination of baculoviruses from the lepidopteran midgut. We discuss whether a similar mechanism might be involved in the dissemination of arboviruses from the mosquito midgut. We also discuss to what extent apoptosis might be part of the mechanisms arboviruses employ to overcome tissue barriers in their mosquito vectors. In order to keep this review concise, we have not included any studies investigating tissue barriers that are responsible for the vertical transmission of arboviruses. Vector immune responses against arboviruses, which also play an important role in determining vector competence, are discussed by Sim *et al.* in another article in this Special Issue.

## 2. Infection Pattern of an Arbovirus in a Competent Mosquito Vector

**Figure 1:**
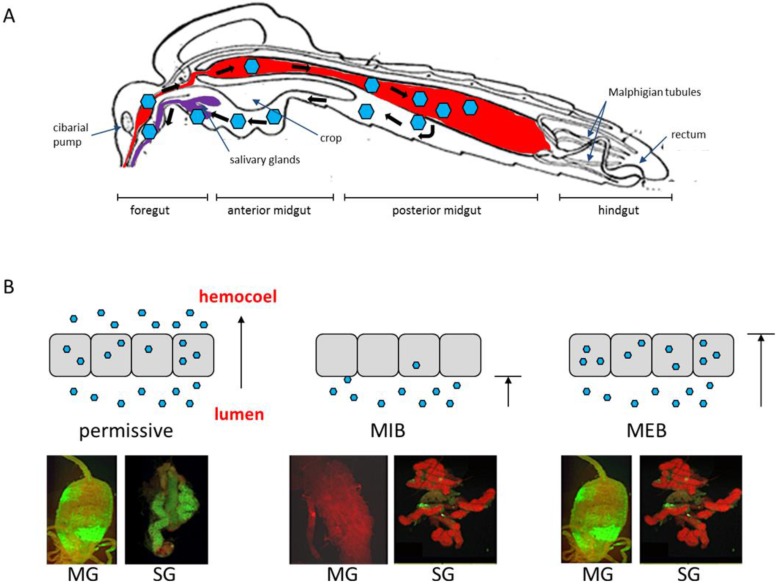
Persistent arbovirus infection of a mosquito vector requires successful crossing of tissue barriers by the virions. (**A**) Schematic representation of arbovirus tropism in a mosquito vector [after Snodgrass [21], modified]. Virions are represented by blue hexagons. (**B**) Schematic representation of a permissive midgut infection, midgut infection barrier (MIB) and midgut escape barrier (MEB). Grey squares represent midgut epithelial cells and blue hexagons represent virions. Images below show the presence of antigen of DENV2-Jamaica 1409 (green) in midgut (at 7 days post-bloodmeal) or salivary glands (at 14 days post-bloodmeal) of *Aedes aegypti*, as detected by immunofluorescence assay using DENV2-specific monoclonal antibody 3H5. Tissues were counter-stained with Evans blue. Images were viewed under a fluorescent microscope equipped with FITC-specific filter sets. MG, midgut; SG, salivary gland.

Vector competence (VC) for an arbovirus is affected by intrinsic (genetic) factors and mechanisms that control the ability of a vector mosquito to acquire, maintain, and transmit an arbovirus. Important VC-related genetic factors are innate immunity-related pathways and tissue barriers in the mosquito that an arbovirus needs to overcome, *i.e.*, the midgut infection barrier (MIB), midgut escape barrier (MEB), salivary gland infection barrier (SGIB), and salivary gland escape barrier (SGEB) (Figure 1). VC-related genetic factors can vary widely between mosquito species and populations or strains. Moreover, only specific arbovirus species and/or strains are adapted to overcome VC-related genetic factors successfully, suggesting that arboviruses and their mosquito vectors are constantly coevolving. As proposed by Hardy and colleagues [15], the key steps for a productive arbovirus infection of a mosquito include (1) initiation of infection in the midgut; (2) spread of infection within the midgut epithelium; (3) dissemination from the midgut to secondary tissues; (4) secondary amplification of the virus in various tissues outside the midgut; (5) infection of salivary glands (and sometimes reproductive tissues for vertical transmission to offspring); and (6) release of the virus into salivary ducts for horizontal transmission to an uninfected vertebrate host. In the following section, we describe the infection pattern of arboviruses in competent mosquito vectors in more detail.

### 2.1. The Midgut as the Initial Site of Infection

**Figure 2:**
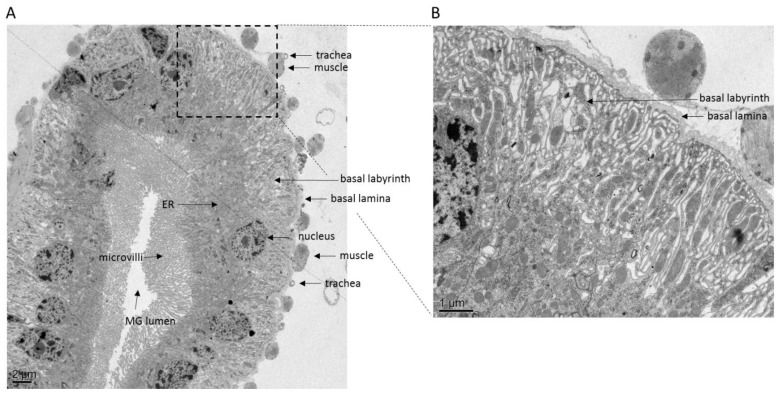
Ultrastructural views of the posterior midgut tissue of *Ae. aegypti*. (**A**) Cross-section of a non-infected midgut of a female at 7 days post-bloodmeal (magnification: 600×). (**B**) Close-up view of image in panel (**A**) at 2000× magnification. Note the structured BL surrounding the midgut. Dissected midguts were fixed in 2% glutaraldehyde, 2% paraformaldehyde fixative followed by embedding in histogel and post-fixation in 1% osmium tetroxide. A dehydration series in ethanol was performed prior to embedding of midguts in Epon/Spurs resin. Resin-embedded midguts were thin-sectioned and stained with lead citrate. Images were captured using a JEOL 1400 transmission electron microscope.

The tube-shaped alimentary canal of mosquitoes can be divided into three major regions, the foregut extending from the mouth, the midgut, and the hindgut extending to the anus [16] (Figure 1A). The mosquito foregut includes the pharynx, esophagus, crop, and the sclerotized proventriculus. The cardiac valve is located at the distal end of the foregut and extends into the midgut. Its function is to prevent contents of the midgut lumen from spilling back into the foregut [16]. The mosquito midgut consists of a single layer of epithelial cells surrounded on the abluminal side by an extracellular matrix termed basal lamina (BL) (Figure 2). The proteinaceous, creased and striated BL encircles the midgut epithelium and separates it from circular and longitudinal muscle cells and trachea, which wrap around the midgut [17]. In the mosquito midgut, a narrow anterior region can be distinguished from a wider posterior region [18]. In the anterior portion of the midgut, which is involved in sugar absorption, cells have prominent microvilli, a smooth endoplasmic reticulum (ER), and a well-developed basal labyrinth, a reticulate network of intercellular spaces located in the basal portion of midgut epithelial cells [19]. The latter is formed by infoldings of the basal plasma membrane. In the posterior portion of the midgut, absorption of blood components is carried out. Here, cells have a prominent rough ER and an elevated number of mitochondria [18]. Acquisition of a bloodmeal drastically changes the epithelial structure of the midgut: The nuclei are condensed whereas mitochondria are enlarged; rough ER whorls appear and accumulation of electron-opaque material has been noted [20].

Following acquisition of a viremic bloodmeal from a vertebrate host, arboviruses enter the midgut lumen [17]. Virions in close proximity to the epithelial cell lining need to enter epithelial cells through the microvilli before the bloodmeal is surrounded by the peritrophic matrix, a chitinous sac secreted by the midgut epithelium into the gut lumen during blood digestion. In *Ae. aegypti*, the peritrophic matrix becomes evident at 4–8 h after bloodfeeding and attains mature thickness and texture by 12 h [22,23]. Infection patterns of midgut epithelial cells vary according to virus–mosquito species combinations. For example, dengue virus type 2 (DENV2) was not detected in the anterior portion of the midgut of *Aedes albopictus*, whereas Japanese encephalitis virus (JEV) infected the entire midgut of *Culex tritaeniorhynchus* [24,25]. During the initial infection of the midgut epithelium, only a small number of cells seem to be receptive to virus infection. Eastern equine encephalitis virus (EEEV) infects around 30% of midgut cells in *Aedes triseriatus* and St. Louis encephalitis virus (SLEV) infects around 20% of *Culex pipiens* midgut cells [26,27]. In a study using radiolabeled virus and reporter gene expressing replicons, Smith and colleagues observed that Venezuelan encephalitis virus (VEEV) infected an average of 28 midgut cells in *Aedes taeniorhynchus* [28]. Initially, many infections occurred in only one to five cells out of ~100 midgut cells that appeared to be receptive to infection with the virus. Similarly, Scholle and colleagues found that ≤15 midgut cells were infected when *Culex quinquefasciatus* mosquitoes ingested replication-incompetent West Nile virus (WNV) virions [29].

**Figure 3:**
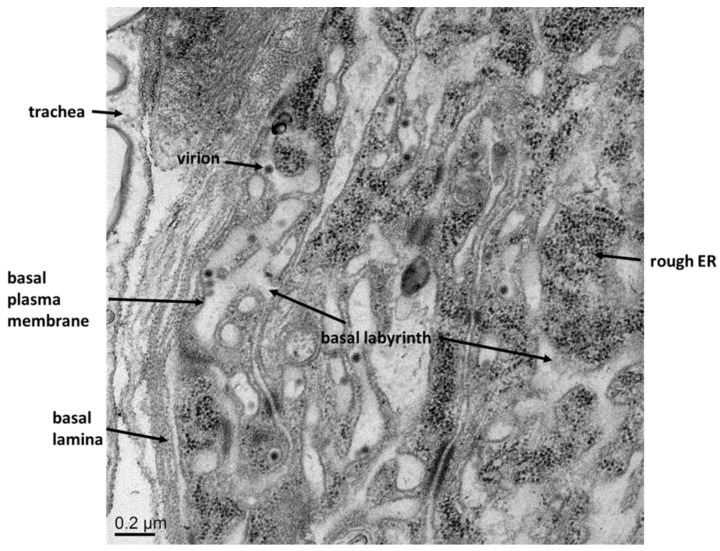
Ultrastructural view of the posterior midgut tissue of an *Ae. aegypti* female orally infected with CHIKV 37997 at 7 days post-infection. Virus titer in the bloodmeal was ~10^7^ pfu/mL. CHIKV virions are present only in the basal labyrinth of the epithelial cell, which is the putative site of viral assembly. Virions are visible budding from the basal plasma membrane in close proximity to the BL. Virions crossing the BL were not observed (magnification: 8000×).

Once the virus has entered a midgut epithelial cell, viral RNA replication takes place at the ER membrane. The site of viral maturation, however, may vary according to virus and mosquito species. In *Cx. pipiens*, *de novo* synthesized virions of SLEV have been first observed within cisternae of the ER followed by subsequent accumulation in the basal labyrinth and in the extracellular spaces between the cell plasma membrane and the BL. Similar observations have been made for EEEV in *Ae. triseriatus* and for western equine encephalitis virus (WEEV) in *Aedes dorsalis* [26,30]. In contrast, WEEV in midguts of *Culex tarsalis* [31] and chikungunya virus (CHIKV) in *Ae. aegypti* (Figure 3) do not appear to enter the cisternae of the ER. Instead, virions are assembled at the plasma membrane lining the basal labyrinth.

### 2.2. Virus Dissemination from Midgut to Secondary Tissues

Current studies strongly suggest that virions need to pass through the BL of the midgut epithelium to enter the hemocoel. Accordingly, an earlier electron-microscopy study showed SLEV virions within a seemingly intact midgut basal lamina of an infected *Cx. pipiens pipiens* female [27]. However, the authors did not describe how the virions localized within the structure of the BL. Similarly, WNV virions have been observed within the BL of *Cx. pipiens* midgut epithelial cells [32]. Apparent changes in midgut BL integrity have been observed in ultrastructural studies of *Culiseta melanura* when infected with EEEV [33]. Within infected anterior and posterior midgut epithelia, degenerate, translucent cells were detected, whose nuclei were vesicular. In these cells the BL was often disrupted and terminated adjacent to degenerating cells. Abnormal BL morphologies following virus infections suggest possible pathways for virus midgut escape.

Following dissemination from the midgut, it has been hypothesized that an arbovirus requires (1) a means to amplify after its escape from midgut epithelial cells, so that it can infect the salivary glands efficiently [15,34], and (2) a vehicle to ensure dissemination from the midgut to the salivary glands [19,35]. Following escape from the midgut, arboviruses typically disseminate to secondary tissues such as fat body, hemocytes, and nerve tissue, whereas only certain viruses, *i.e.*, VEEV and WNV, have been found to also infect muscle tissue [34,36]. Hemocytes could indeed form an important continuum between midgut tracheoles and the salivary glands [35]. Of the four types of mosquito hemocytes, prohemocytes, plasmatocytes, oenocytoids, and the most abundant, phagocytic granulocytes, the latter were the most common ones in *Ae. aegypti* to be infected with SINV [37]. It was concluded that hemocyte infection might be an important amplification step for arboviruses outside the midgut before the virus is infecting the salivary glands. Once the salivary glands of the mosquito become infected, virus is transmitted along with the saliva.

### 2.3. Infection of Salivary Glands

Mosquito salivary glands are paired organs located in the thorax. Each gland consists of three lobes (two lateral lobes and one wider, shorter median lobe) connected to a main salivary duct [38]. The lateral lobes can be divided into proximal and distal regions. Different regions of the glands excrete different proteins. The proximal lateral lobes produce enzymes involved in sugar feeding, whereas the median and distal lateral lobes produce lectins and proteins such as Apyrase and D7, which aid the bloodfeeding process by degradation of adenosine diphosphate, and interference with hemostatic and vertebrate host immune responses, respectively [39]. Each lobe has a central internal duct encircled by a monolayer of epithelial cells bounded externally by a BL [38]. Arbovirus infection of salivary glands typically begins in the distal lateral lobes [40,41,42,43,44]. Certain viruses such as DENV2 and CHIKV infect the proximal lateral and median lobes of *Ae. aegypti*, whereas Sindbis virus (SINV) does not seem to infect the median lobe in *Ae. albopictus* or *Ae. aegypti* [40,41,45,46]. Thus, Salazar and colleagues speculated that the distal lateral lobes of salivary glands in aedine mosquitoes are the site containing receptors to enable endocytosis of arboviruses [41]. Replication of flaviviruses is thought to occur at smooth membrane structures of the salivary glands, as shown by the presence of the replicative form of viral RNA and nonstructural proteins involved in RNA replication of WNV in *Cx. quinquefasciatus.* Proteolytic processing of WNV, and other flaviviruses, likely occurs in association with virus-induced convoluted membranes and tubular proliferated membranes, which are hypothesized to be alternate and reversible structures [47,48,49,50]. Following replication, virus is deposited into the apical cavities of acinar cells, which can lead to inoculation into a host upon refeeding [51]. Mosquito saliva contains complex protein peptide mixtures such as sugar degrading enzymes (glycosidases), antimicrobials, anti-hemostatics, proteins with angiogenic or anti-inflammatory properties, and immune modulators, which are injected into the vertebrate host along with virions [52].

## 3. Dose-dependent and -independent Tissue Barriers to Infection

Factors that strongly affect VC of a mosquito for a particular arbovirus include MIB, MEB, SGIB, and SGEB [53] (Figure 1B). These barriers can be virus dose-dependent and/or -independent [15]. Dose-dependent tissue barriers can be affected by innate antiviral responses in the mosquito such as the RNA interference (RNAi) pathway [54]. RNAi limits the replication efficiency of SINV in *Ae. aegypti* and impairment of the RNAi pathway allowed the virus to more rapidly and efficiently overcome its dose-dependent midgut infection and escape barriers [54]. Kramer and colleagues were the first to demonstrate that the inability of infected *Cx. tarsalis* mosquitoes to transmit WEEV was associated with a MEB [55]. In female mosquitoes exhibiting a MEB, virus infected the midgut but was unable to disseminate to other organs, remaining sequestered in the infected midgut cells. This MEB was found to be dose-dependent and occurred only when low doses of virus had been ingested.

Dose-independent barriers to arbovirus infection may involve incompatibilities between the virion and tissue surface structures, preventing the virus from binding to and/or traversing tissues. A particular mosquito strain can exhibit tissue barriers to several arboviruses and a particular virus strain can encounter infection barriers in various mosquito strains. Thus, arbovirus populations encounter bottlenecks in their natural vectors during infection, dissemination, and transmission as shown for viruses such as WNV, VEEV, and CHIKV in their associated mosquito vectors (reviewed in [51]). Accordingly, these bottlenecks occur at the midgut level (infection/dissemination) and at the salivary gland level (infection/release of virions with saliva) [56].

## 4. The Midgut Infection Barrier

MIB for arboviruses have been described for decades [15,31,57,58,59] (Figure 1B). A MIB can result from two different events: (1) the virus is unable to invade epithelial midgut cells, or (2) the virus is able to invade but is unable to replicate or spread to other cells. The latter scenario could occur following a strong immune response that blocks virus replication. Franz and colleagues showed that the mosquito antiviral RNAi pathway can be primed to establish a complete MIB for DENV2 following cell entry and causing mosquitoes to be refractory to the virus [14,60]. The other mechanism leading to a MIB involves the refractoriness of midgut epithelial cells to arbovirus infection. Several hypotheses have been proposed to explain this phenomenon: (1) diversion of the blood and virus into the ventral diverticulum or crop (a chitin-lined sac used for carbohydrate nutrient storage); (2) filtration of virus by the peritrophic matrix [61]; (3) inactivation of virions by midgut digestive enzymes; (4) virus-midgut cell charge interactions that preclude binding; and (5) the absence of appropriate receptors on the apical surfaces of epithelial cells. Hypotheses (1) to (4) do not have experimental support [15,62], whereas hypothesis (5) has been supported by several studies, as described in the following sections.

### 4.1. MIB for Alphaviruses

It is generally assumed that most arboviruses enter midgut epithelial cells via receptor-mediated endocytosis. However, alphaviruses may be able to enter midgut epithelial cells through either endocytosis or direct penetration [63]. Some alphaviruses, such as Semliki Forest virus (SFV) and WEEV, have been observed to uncoat their RNA after direct fusion of the virion envelope and the microvillar membrane of the midgut cell instead of releasing their viral genomes in vesicles as with most arboviruses [30,64]. It has been hypothesized that the interaction of virus proteins with a receptor could allow conformational changes that lead to the formation of a protein pore-like structure to penetrate the plasma membrane.

For alphaviruses, the only midgut infection determinant identified to date is the E2 envelope glycoprotein, which forms spikes on the virion surface [65] and probably interacts with cellular receptors [66,67]. Based on saturation and competitive binding studies, Houk and colleagues provided evidence that the binding of WEEV to the brush border (the microvillar surface of the gut) in susceptible *Cx. tarsalis* appeared to be highly specific; in contrast, binding to the brush border from refractory *Cx. tarsalis* and *Cx. pipiens* strains was nonspecific [57]. Based on the specific binding data, it was estimated that the brush border from susceptible *Cx. tarsalis* yielded an estimated 1.8 to 3.5 × 10^6^ binding sites per mesenteronal epithelial cell with an affinity constant of (Ka) of 2.2 × 10^11^ × M^−1^.

Several attempts have been made to identify potential mosquito midgut receptors for alphaviruses. Strauss and coworkers used monoclonal antibodies to identify a 67 kDa protein with high laminin affinity as a putative alphavirus receptor in mammalian and mosquito cells [68]. Similarly, a laminin-binding protein with a mass of 32 kDa was identified as a putative receptor for VEEV in mosquito cells [69]. Mourya and colleagues identified two proteins of 38 and 60 kDa from brush border membranes of *Ae. aegypti* midguts that were linked to susceptibility to CHIKV infection [70]. These proteins were found to be in lower concentrations in refractory than in susceptible strains. It is tempting to speculate that these proteins correspond to the 32 and 67 kDa proteins discovered earlier [68,69]. However, in a later study it was suggested that alphavirus affinity for laminin receptors was likely an artifact of cell culture adaptation of viruses, which also increased their affinity to heparin sulfate by rapid acquisition of envelope glycoprotein mutations [71]. Recently, isolated alphaviruses showed a reduced affinity for heparin sulfate structures. Instead, alphaviruses, like flaviviruses, used pattern recognition receptor proteins of the C-type lectin family (Ca_2_^+^-dependent carbohydrate binding proteins) such as DC-SIGN and L-SIGN (containing high amounts of mannose) as attachment receptors in the mosquito vector [71,72]. In addition, Rose and colleagues described the divalent metal ion transporter natural resistance-associated macrophage protein (NRAMP) as a potential receptor for SINV [73].

### 4.2. MIB for Flaviviruses Based on Studies with DENV

Earlier studies suggested that the MIB is a major determinant of vector competence for flaviviruses such as DENV [74,75]. Thus, differences in mosquito vector competence may, in part, be due to arbovirus-specific midgut epithelial receptors [76]. Domain III of the flavivirus envelope (E) protein interacts with cell surface structures (*i.e.*, receptors) of the mosquito vector, thereby determining vector competence at the midgut epithelial cell entry level [77]. Numerous studies have attempted to identify the DENV receptor complex in mosquito cells, resulting in the identification of several proteins or glycoproteins which could serve as receptors. Previously-reported low affinity heparan sulfate interactions with flaviviruses are not thought to be a true receptor-mediated process. Instead, it is considered as a non-specific mechanism to concentrate virions on the cell surface [78]. In contrast, antibodies against 70 kDa and 95 kDa proteins in *Ae. albopictus* C6/36 cells efficiently blocked WNV, JEV, and DENV entry, suggesting that these proteins form part of a receptor complex for mosquito-borne flaviviruses [79]. Antibodies against an 80 kDa protein in C6/36 cells [80] and antibodies against 67 and 80 kDa proteins, located at the apical membrane of the midgut epithelium in *Ae. aegypti*, inhibited DENV1-4 infection [81]. In another study, utilizing three different *Ae. aegypti* strains with different levels of vector competence for DENV2, the 67 kDa protein responsible for enabling infection of midgut cells could be used as a marker of vector competence for DENV [76]. It was also suggested that laminin-binding proteins of 37/67 kDa and/or 50 kDa may be involved in DENV2, DENV3 and DENV4 infection of C6/36 cells [82]. Further, DENV4 was shown to bind to two glycoproteins of 40 kDa and 45 kDa that are present on the surface of C6/36 cells. Both proteins appeared to be immunologically related to Hsp 90 [83,84]. Later, Kuadkitkan and coworkers identified a ubiquitously expressed protein, prohibitin, which acted as a receptor for DENV2 to facilitate its internalization into mosquito cells [85]. Curiously, the protein interaction was specific only for the E protein of DENV2 and not for other flaviviruses. In another experimental approach, Muñoz and colleagues identified midgut proteins that specifically bound to DENV virions and the recombinant domain III of DENV E protein. The bound proteins had apparent molecular masses of 57, 67, and 80 kDa [86]. Following purification and mass spectrometric analysis, enolase (57 kDa and 67 kDa), beta-adrenergic receptor kinase (beta-ARK; 67 kDa), translation elongation factor EF-1 alpha/Tu (57 kDa), and cadherin (80 kDa) were identified. Enolase has been found to localize in the midgut brush border and has been suggested to be a receptor for DENV2 in *Ae. aegypti* midgut cells. Furthermore, enolase may correspond to the 67 kDa protein previously reported [76,81].

It can be concluded that the nature of MIB preventing entry of arbovirus virions to midgut epithelial cells is still far from being fully understood. A wide array of studies using various techniques identified a range of proteins, many of which potentially play a role as surface receptors for cell entry of alphaviruses and flaviviruses. Considering the various studies it becomes obvious that cell surface receptor-virion interactions are often highly strain/species specific. Furthermore, it is likely that arbovirus receptors consist of protein complexes, instead of single proteins. Based on the observations described above, the density of midgut receptors relevant for virus cell entry can vary substantially between susceptible and refractory mosquito strains. It is also possible that in mosquito strains exhibiting a strong MIB for a particular arbovirus, protein conformations of the relevant receptor(s) differ (due to polymorphism) in such a way that the virus can no longer utilize the receptor(s) for cell entry.

## 5. The Midgut Escape Barrier

Kramer and colleagues demonstrated that the inability of infected *Cx. tarsalis* mosquitoes to transmit WEEV was associated with a dose-dependent MEB, which may be associated with a genetically controlled mechanism that modulates replication of arboviruses in the mosquito midgut [55]. Accordingly, Bennett and colleagues were able to map quantitative trait loci (QTL) for DENV2 dissemination in *Ae. aegypti* in an advanced intercross line based on a hybrid between a “high dissemination” and a “low dissemination” strain [87]. As a result, three QTL positions were identified: *Riboptl1* (ribosomal protein L1), *TrypAbun* (abundant trypsin), and *AspSyn* (asparagine synthetase) on chromosome I, II and III, respectively.

In addition to dose-dependent MEB, dose-independent MEB have also been described for specific virus-vector combinations such as Rift Valley fever virus (RVFV) in *Cx. pipiens* and VEEV in *Culex taeniopus* [88,89]. It seems plausible that a dose-independent MEB involves the structure of membrane components separating the midgut tissue from the hemocoel and other structures such as the BL, which the virus cannot efficiently traverse.

### 5.1. The Basal Lamina, a Physical Barrier for Virions

The BL is a proteinaceous matrix secreted by epithelial cells on their basolateral surface [90]. BL provides structure to the epithelium as it is continuously being renewed after injury and also provides separation of organismal compartments or cell layers. Its porous composition serves as a filter, allowing permeation by molecules but likely blocking passage of larger microorganisms. BL are densely packed with cross-linked proteins, mainly collagen type IV, laminin, perlecan, and entactin. In mosquitoes, the BL varies in thickness and structure. In large *Ochlerotatus triseriatus* females, the BL surrounding the midgut consists of nine to 16 layers, but in small, undernourished females it can be only three to six layers [91]. Following oral challenge with La Crosse virus (LACV), small females exhibited no MEB whereas 31% of large females showed strong MEB. Thus a thinner BL may allow more rapid dissemination of the virus from the midgut, supporting the conclusion that the BL surrounding the midgut must be traversed by arboviruses during dissemination [91,92]. However, differing BL thickness amongst *Ae. albopictus* strains was not a determinant of DENV1 dissemination [92]. As only a small number of infectious virions seem to be needed to mount a systemic infection in the vector, any small breach of the BL should allow effective virus dissemination to secondary organs. During blood feeding, the mosquito gut expands in diameter several-fold, and then slowly returns to its pre-feeding state over the next few days. During this period of expansion and contraction, it seems likely that extensive reorganization of the midgut BL must occur. It is possible that transient gaps in the BL caused by degradation and re-synthesis of BL components are exploited by arboviruses, allowing them to cross into the tracheal system and/or the hemocoel. However, the observation of virus strain- or vector-specific MEB argues against passive passage of virions through a transiently leaky BL as a general midgut escape mechanism. Alternative, possible mechanisms explaining arbovirus midgut escape are described in the following sections.

### 5.2. The Role of Tracheae in Virus Dissemination from Midgut

Romoser and colleagues considered the long-standing question whether/how arboviruses are able to bypass the non-cellular BL of the midgut a key issue in arboviral pathogenesis in mosquitoes [19]. Several arboviruses from different virus families including DENV, WEEV, SINV, LACV, and RVFV, as well as the insect pathogenic baculoviruses, have been observed to infect tracheal epithelial cells following midgut cell infections of their mosquito vectors or lepidopteran hosts [15,30,31,40,41,55,93,94,95,96,97,98,99,100]. Thus, in at least some virus-vector combinations, dissemination of arboviruses from their primary site of infection, *i.e.*, midgut epithelial cells, likely occurs via the tracheal system surrounding the midgut tissue [19,97]. Tracheae form a network of continuous tubular channels, which communicate with the external environment via openings (spiracles) in the integument and are responsible for oxygen supply to the various tissues [19]. The spiracles open into large tracheae that branch and become progressively smaller, ultimately ending in a single cell, the tracheoblast of epithelial origin containing one or more open chitinous end tubes, the tracheoles. Midgut tracheae are closely associated with visceral muscle fibers, which are incorporated in an outer layer of the BL. This outer layer is seemingly penetrated by the tracheoles to oxygenate midgut epithelial cells, even though earlier ultrastructural studies suggested that the BL might not be completely penetrated [18].

Reporter gene-expressing VEEV replicon particles, which were capable of only a single round of infection, were observed to infect only midgut epithelial cells when orally acquired by *Ae. taeniorhynchus*, and not any cells outside of the midgut [19]. Moreover, when VEEV replicon particles were intrathoracically injected, there was infection of muscles and tracheae surrounding the midgut, but no infection of the midgut epithelium. On the contrary, the midgut epithelium became infected when replication-competent VEEV was intrathoracically injected. These results support the idea that the virus requires replication in order to access some sort of conduit and cross the midgut barrier, which includes BL having a size exclusion limit below the size of arbovirus virions. These conduits were postulated to be either tracheae or tracheoblasts that penetrate into the BL, or regions of modified “spongy” BL associated with visceral muscle fibers, which are in contact with midgut epithelium [19,94,97]. Virions may be able to enter and infect the tracheo-muscular complex where the “spongy” matrix is present, which resembles the matrix material of the proventriculus, but crossing the barrier requires replication.

Based on these findings, Romoser and colleagues proposed a working model of arbovirus dissemination [19]. Accordingly, virus ingested with a bloodmeal infects one or more midgut epithelial cells, replicates and subsequently enters the basal labyrinth of the epithelial cells. From this point onwards virus could (1) infect adjacent epithelial cells, thereby spreading and amplifying within the midgut epithelium; (2) infect the cytoplasm of a tracheal cell and bypass the unmodified BL; or (3) escape via the matrix of the modified BL and infect the tracheo-muscular complex, or perhaps bud directly into the hemolymph from a muscle cell.

### 5.3. How Baculoviruses Escape from the Lepidopteran Midgut—A Model for Arboviruses?

Budded virions of baculoviruses, large DNA-containing viruses that infect insects, are able to cross the BL surrounding the lepidopteran midgut epithelium by either passing through gaps in the BL formed due to normal BL component turnover or, more rapidly, by directly stimulating signaling events that degrade proteins in the BL of tracheoblasts adjoining the midgut BL (reviewed in [99]). Following infection of midgut cells with a baculovirus, progeny virions have sometimes been observed in the lepidopteran hemocoel within a short time that is not long enough for virus replication to have occurred in midgut cells. Thus, it was hypothesized that baculoviruses can cross the midgut cell BL prior to virus replication, with some of the infecting virions crossing directly into the hemocoel [101]. Virions were also observed adjacent to the midgut cell BL. However, the direct budding of virions from the midgut across the BL and into the hemocoel has not been observed so far.

Baculoviruses have also been shown to infect tracheal cells overlaying and most likely invading the midgut BL. The observation that these tracheal cells were the first identifiable cells to be infected after the midgut cells led to the proposal that the tracheal system, linking the midgut and organs in the hemocoel, allowed passage of the virus between these two BL-separated compartments [94]. Further support for tracheal cells serving as an escape route into the hemocoel came from the observations that a baculovirus-encoded fibroblast growth factor (FGF) caused delamination of tracheal cell BL and this facilitated systemic spread of baculoviruses [102]. The viral FGF homolog triggered, directly or indirectly, the activation of lepidopteran MMPs in the extracellular matrix of midgut cells. MMP activation was required for activation of secreted effector caspases that were in turn responsible for the degradation of BL proteins, disrupting the architecture of tracheal BL and enabling viruses to infect the tracheal cells. It was hypothesized that the viral FGF functions by stimulating tracheal cell motility, which is the function of FGF in *Drosophila*, and triggering tracheoblast motility, leading to degradation and re-synthesis of tracheal cell BL and transiently allowing virus accessibility to tracheal cells.

An important question is whether a similar signaling pathway involving FGF, MMPs and caspases may be also activated in the mosquito midgut in the presence of replicating arboviruses to remodel the BL for virus dissemination. Thus, it may be possible that after blood feeding, a FGF-MMP signaling pathway helps to remodel the BL associated with tracheae or the tracheo-muscular complex.

### 5.4. Dissemination via Cardia/Intussuscepted Foregut

Apart from the tracheae, which are seen as a critical site for arbovirus dissemination from the mosquito midgut, the cardia/intussuscepted foregut region has been considered another possible site for virus dissemination [103,104]. The cardia is an esophageal invagination located at the foregut-midgut junction, adjacent to the intussuscepted foregut. Similar to the tracheal/muscle complex, the cardia is associated with a matrix, which has a “spongy” texture of modified BL [104]. Like midgut epithelial cells, the cardia contains a network of tracheae and is associated with muscle fibers. When studying the dissemination of RVFV in *Cx. pipiens*, it was observed that the most common sequence of tissue/organ infection following dissemination of the virus from the midgut epithelium was: Intussuscepted foregut, fat body, salivary glands and thoracic ganglia, epidermis, and ommatidia of the compound eyes [104]. Further, orally acquired RVFV virions were observed in the basal labyrinth associated with the outer cardial epithelial cells and in the matrix associated with the inner cardial epithelial cells before being detected in cells of the intussuscepted foregut. In mosquitoes that were infected with RVFV via intrathoracic injection, the sequence of infection was reversed: Viral antigen was first detected in the cells of the intussuscepted foregut and later in the cardial epithelial cells [104].

Following oral acquisition of an epidemic strain of VEEV by *Ae. taeniorhynchus*, the virus was initially detected in the posterior midgut and, occasionally, in the anterior midgut including the cardia [59]. Dissemination of VEEV was detected at two days post-infection, at the same time when the intussuscepted foregut was first observed to contain viral antigen. It was speculated that the virus was able to spread cell-to-cell from the cardia to the adjacent intussuscepted foregut, thereby disseminating into the hemocoel without traversing the BL. However, in some mosquitoes, disseminated infections occurred before the intussuscepted foregut was infected, indicating that the virus may also use the posterior midgut to disseminate to secondary tissues. Once dissemination beyond the midgut occurred, virus was present in nearly all tissues. Similarly, a high correlation between WEEV infection of the cardia and dissemination efficiency into the salivary glands could be established for highly competent mosquito strains of *Cx. tarsalis* but not for less competent mosquitoes [98]. A conflicting observation was made when orally infecting *An. gambiae* with different recombinant ONNV strains [105]. Occasionally, mosquitoes showed strong infection of the cardia while infection of the posterior midgut was limited to small foci. Overall dissemination from the midgut was <30% for all ONNV strains tested. This prompted the authors to speculate whether ONNV infection of the cardia was an artifact resulting from artificial bloodfeeding. However, when DENV3 was intrathoracically injected into non-bloodfed *Ae. aegypti*, the intussuscepted foregut and cardia were among the initial tissues infected with the virus [106]. Other examples where the cardial epithelium/intussuscepted foregut become infected include JEV, which was orally acquired by *Cx. tritaeniorhynchus*, *Cx. vishnui*, and *Cx. pseudovishnui* [25], and recombinant SINV-MRE16 which was orally acquired by *Ae. aegypti* [100,107].

To summarize, tissues that have always been observed to be infected in competent arbovirus-mosquito vector combinations include posterior midgut epithelium, trachea, fat body, hemocytes, nervous tissue, and salivary glands. Tissues and organs that are sometimes, but not always, infected include the longitudinal muscle tissue, Malphigian tubules, and the cardia/intussuscepted foregut region. Specifically, the role of cardia/intussuscepted foregut infection in facilitating efficient dissemination of arboviruses from the mosquito gut to the salivary glands remains unclear at this point, but it may serve as a conduit for midgut escape in certain arbovirus-mosquito combinations.

## 6. Salivary Gland Infection and Escape Barriers

Other barriers to arbovirus transmission that have been described in arthropods are SGIB and SGEB. A dose-dependent SGIB has been demonstrated for WEEV in *Cx. tarsalis* [55] and descriptions of SGIB phenomena (either dose-dependent or dose-independent) have been made for various virus-mosquito strain combinations (LACV in *Ae. triseriatus*, EEEV in *Ae. albopictus*, and JEV and WNV in *Cx. pipiens* [108,109,110].

Molecular mechanisms that clearly explain SGIB or SGEB for viruses in arthropods have not been described so far. Scott and colleagues showed that the SGIB for EEEV was not dependent on infection of fat body surrounding the midgut and salivary glands in mosquitoes [109]. The virus disseminated from the midgut and infected fat body but was unable to enter the salivary gland tissue or to establish infection in the salivary glands after entry. Romoser and colleagues suggested that the BL surrounding the salivary glands may be the major barrier affecting the SGIB for RVFV in *Aedes spp.* [97]. Furthermore, the authors suggested that the structure of the BL may occasionally obscure cell surface receptors necessary for cell entry of a particular arbovirus. To explain the SGIB for baculovirus in *Bombyx mori*, the tracheal system organization and the presence of the BL surrounding the salivary glands were hypothesized as major obstacles for the virus, essentially preventing the penetration of budded virions into the salivary glands [111]. Hardy and colleagues speculated that hemolymph could be an important determinant of the SGIB, as virus titers in the hemolymph of *Cx. tarsalis* susceptible to WEEV were significantly higher than in those of refractory females [15]. In this case productive infection of hemocytes might have been limited; however, this was not analyzed in the study.

Barreau and coworkers observed that various monoclonal antibodies bound differentially to the lateral and median lobes of *Ae. aegypti* salivary glands, documenting region-specific biochemical differences on salivary gland surfaces [112]. Later, expression of 30 endogenous transcripts in the salivary glands of *Ae. aegypti* were spatially mapped, revealing unique localization patterns [113]. It was observed that certain salivary gland transcripts mapped exclusively to the median lobe or the lateral lobes, while others were expressed in both the median and lateral lobes. The authors speculated that the differences in transcription profiles of lateral and median lobes account for the difference in susceptibility to virus infection. In the internal ducts of the lateral lobes, cuticular filamentous membrane extensions were detected, which are absent in the median lobe of the salivary glands [46]. These membranous extensions contained heparan-sulfate proteoglycan (HSPG). Studies comparing SINV strains revealed that SINV-AR339 was HSPG-dependent and also caused strong cytopathic effects (CPE) in lateral lobes, whereas SINV-TR339 was not HSPG-dependent and did not cause significant pathology. It was concluded that HSPG might act as an attachment factor for certain arboviruses within the salivary gland ducts. Ciano and colleagues suggested that prominent cytopathic effects in the lateral lobes allows for virus release [46], whereas HSPG could act as a sponge to retain virus in the lateral lobes. Earlier, it was hypothesized that transmissible virus originates in the duct wall of mosquito salivary glands [114]. In this case, destruction of gland acinar cells (consisting of epithelial cells surrounding a lumen where the saliva is deposited after being produced by the secretory cells) via induction of apoptosis, while leaving internal ducts intact, might be necessary to release virus along with saliva. For this to occur, viruses would need to induce apoptosis in the salivary glands to be transmissible; however there are not sufficient data available to support this. SGEB have been previously reported for LACV and SINV transmission by *Aedes* and *Culex* species [115,116,117,118]. For example, *Aedes hendersoni* was shown to be an incompetent vector of LACV because of a SGEB in which the salivary glands were infected but the mosquito failed to transmit the virus orally [116]. More recently, SGEB have been also reported for RVFV [97,119].

## 7. What Role Do RNAi and Apoptosis Play in Barriers to Infection?

In several studies, it was shown that the antiviral RNAi pathway can affect dose-dependent MIB and MEB in *Ae. aegypti* [14,54,60,120,121]. Transgene-mediated triggering of RNAi against DENV2 can result in a complete MIB for the virus, which eliminated the virus from this primary tissue [14]. Moreover, RNAi impairment in *Ae. aegypti* caused dramatic increase of arbovirus titers in midgut tissue, enabling viruses to disseminate more rapidly from the midgut to secondary tissues. As a consequence, the extrinsic incubation period in the vector was shortened by several days [122].

The possible role of apoptosis in overcoming tissue barriers to arbovirus infection is not well understood. Presumably, there is a constant low level of apoptosis occurring in midgut, as homeostasis of the midgut is a continuous process of death and replacement of midgut epithelial cells. One question to consider is whether increased apoptosis, stimulated by arbovirus infection, could result in the virus breaching the BL. In most compatible vector/arbovirus relationships, there is little or no apoptosis induction by the virus in mosquitoes. However, there have been reports of cytopathology following arbovirus infection of midgut or salivary glands in certain situations, suggesting that apoptosis could be more common in non-compatible vector/arbovirus combinations. Girard and colleagues reported increased apoptosis in salivary glands of *Cx. pipiens* infected with WNV, and this appeared to correlate with reduced transmission over time [47]. An increase in apoptosis has also been reported in salivary glands of *Ae. albopictus* infected with SINV [123]. An *in situ* TUNEL assay revealed that SINV-infected apoptotic cells were scattered throughout the proximal and distal regions of the salivary gland lateral lobes but were not detected in the median lobe. Curiously, apoptosis was also identified in salivary gland duct cells in both infected and uninfected mosquitoes.

In another example, apoptosis was observed in midgut cells of a WNV-resistant *Cx. pipiens* strain. However, it remains unclear whether apoptosis is the reason for the resistance to infection observed in this strain [124]. Directly testing the effects of apoptosis on arbovirus infection requires experimental manipulation of apoptosis in infected cells of the mosquito. Two approaches have been used to do this during SINV infection of *Ae. aegypti* mosquitoes: (1) using RNAi to induce or inhibit apoptosis during infection, and (2) expressing pro-apoptotic genes from the SINV genome. In the former approach [125], apoptosis was stimulated in mosquitoes by using RNAi to silence expression of AeIAP1, an anti-apoptotic protein that is required to prevent spontaneous apoptosis in *Ae. aegypti* cells [126]. Stimulating widespread apoptosis resulted in death of the majority of the mosquitoes within 1–2 days after injection of *Aeiap1* dsRNA [125]. When midguts of surviving mosquitoes were examined histologically at three days after dsRNA injection, there was evidence of apoptosis and altered midgut morphology. It is perhaps not surprising, then, that oral infection of mosquitoes in which *Aeiap1* had been silenced resulted in higher levels of SINV infection in the midgut and also in the eyes and salivary glands than infection of mosquitoes that had been injected with control dsRNA [125]. This result suggests that high levels of apoptosis may have allowed increased midgut dissemination rates; however, the levels of midgut apoptosis in this experiment were likely higher than during an infection. Further, other antiviral defenses such as RNAi may have been negatively affected by high levels of apoptosis prior to infection. Interestingly, when the initiator caspase AeDronc, which plays an important role in apoptosis in *Ae. aegypti* cells [126], was silenced by RNAi, there was a negative effect on SINV infection of the midgut, and also less dissemination to the eyes and salivary glands [125]. This result suggests that some level of apoptosis, or at least caspase activity, is required for optimal SINV infection and dissemination. It may be that effector caspases are involved in modifying the BL of the midgut or midgut-associated tracheal cells, as was observed during baculovirus midgut escape [99].

In the second approach, the *Drosophila* pro-apoptotic gene *reaper* was inserted into a SINV infectious clone under control of a viral promoter, and this virus was used to orally infect *Ae. aegypti* mosquitoes [127]. Thus, in contrast to the first approach, apoptosis was only stimulated in infected cells. The SINV clone expressing Reaper infected mosquitoes less efficiently and initially replicated to lower titers than control viruses. In addition, strong selective pressure was observed against expression of Reaper in the progeny virus collected from the mosquitoes, indicating that apoptosis has a strong negative effect on the ability of SINV to replicate in the mosquito [127]. Thus, based on this result, it would seem unlikely that apoptosis plays a direct role in virus dissemination. It is still possible, though, that caspases are involved in midgut escape in a non-apoptotic capacity.

## 8. Summary and Conclusions

The findings discussed in this review show that arboviruses are confronted with several tissue barriers during establishment of persistent infection of a mosquito vector. Being competent to acquire, maintain, and transmit an arbovirus requires co-evolution between virus and vector. Major barriers to systemic infection are MIB, MEB, SGIB, and SGEB. The MIB is likely based on receptor recognition as a prerequisite for successful endocytosis of a virus into midgut epithelial cells. If the virus-vector combination is not compatible, the midgut epithelium cannot be infected by the virus, which is then excreted from the organism. Interestingly, even in compatible situations only a very small proportion of midgut cells appear to be susceptible to virus infection. Available evidence suggests that the BL, with its size exclusion limit below the size of virions, is the critical structure making up the MEB. Several mechanisms of midgut dissemination of arboviruses have been described, including via trachea/muscular complex or via the intussuscepted foregut, where the midgut BL may be porous and disordered. However, even in these regions where the BL may be more porous, virus replication often seems to be necessary for midgut escape, and the reasons for this are not understood. Alternatively, a signaling pathway observed for baculovirus dissemination from the lepidopteran midgut has been hypothesized to be involved in arbovirus dissemination. Accordingly, FGF- or possibly other signaling molecules, as well as MMPs and effector caspases may be activated in the presence of replicating arboviruses to remodel the BL so that virions are able to pass through it. This hypothesis is at the center of a current major research effort by the authors.

Following successful dissemination from the midgut, arboviruses typically infect hemocytes, fat body, neural tissue, and occasionally muscle tissue before infecting the salivary glands. Virus amplification in fat body and specifically in hemocytes has been regarded as an important prerequisite before the virus is ready to infect the salivary glands. For certain arbovirus strain-mosquito strain combinations, SGIB and SGEB have been described. Invasion of salivary glands, similar to midgut infection, is likely receptor-mediated. Release of virus-containing saliva via salivary ducts of the infected glands might be dependent on apoptosis, which may be triggered by the virus to disrupt the acinar cells of the glands. The role of apoptosis in arbovirus infection of mosquitoes is still not very clear. Experimental data suggest that short-term induced apoptosis may be required by arboviruses to escape from the midgut and/or to be efficiently released into salivary ducts. However, a recent study clearly demonstrated that there is strong selection against constitutively-induced apoptosis in arbovirus-infected mosquito cells, which appears to have detrimental effects for the virus and also for the vector.

This review examines arbovirus transmission by mosquitoes predominantly from the vector perspective. Obviously, there must also be viral determinants that allow for efficient midgut infection/escape and salivary gland infection/release via saliva. It is assumed that virion surface structures are mainly responsible for efficient crossing of these tissue barriers as described in several studies [128,129]. For example, Khoo and colleagues described a single charge-changing amino acid substitution in the E protein of DENV2-QR94, a virus confronted with a strong MEB in *Ae. aegypti*, to allow efficient escape from the midgut [130].

Even though various concepts regarding the properties and functions of tissue barriers to arboviruses have been discussed for decades, this review highlights that a detailed understanding of these barriers at the molecular and biochemical levels is still lacking. Specifically, previously identified receptor molecule candidates, which seem to be associated with MIB for arboviruses, need to be experimentally validated in mosquito vectors. The molecular nature of MEB including the biochemical pathway underlying BL remodeling in mosquitoes has yet to be resolved. Furthermore, the molecular mechanisms behind SGIB and SGEB and the role of apoptosis in the latter are still not understood. To further understand and develop these concepts, several methodologies based on imaging, molecular, biochemical, and genomic analyses need to be pursued. Fortunately, molecular and genetic tools have recently become available to pursue these studies in more detail in mosquitoes.

## Abbreviations

CHIKV: chikungunya virus (*Alphavirus*, *Togavirirdae*)
DENV1–4: dengue virus serotypes 1–4 (*Flavivirus*, *Flaviviridae*)
EEEV: eastern equine encephalitis virus (*Alphavirus*, *Togavirirdae*)
JEV: Japanese encephalitis virus (*Flavivirus*, *Flaviviridae*)
LACV: La Crosse virus (*Orthobunyavirus*, *Bunyaviridae*)
ONNV: O’nyong-nyong virus (*Alphavirus*, *Togavirirdae*)
RVFV: Rift Valley fever virus (*Phlebovirus*, *Bunyaviridae*)
SFV: Semliki Forest virus (*Alphavirus*, *Togavirirdae*)
SINV: Sindbis virus (*Alphavirus*, *Togavirirdae*)
SLEV: St. Louis encephalitis virus (*Flavivirus*, *Flaviviridae*)
VEEV: Venezuelan equine encephalitis virus (*Alphavirus*, *Togavirirdae*)
WEEV: western equine encephalitis virus (*Alphavirus*, *Togavirirdae*)
WNV: West Nile virus (*Flavivirus*, *Flaviviridae*)

## References

[1] Higgs S., Beaty B.J., Marquardt W.C. (2005). Natural cycles of vector-borne pathogens. Biology of Disease Vectors.

[2] Blair C.D., Adelman Z.N., Olson K.E. (2000). Molecular strategies for interrupting arthropod-borne virus transmission by mosquitoes. Clin. Microbiol. Rev..

[3] Kuno G., Chang G.J. (2005). Biological transmission of arboviruses: Reexamination of and new insights into components, mechanisms, and unique traits as well as their evolutionary trends. Clin. Microbiol. Rev..

[4] Beaty B.J., Bernhardt S., Black W., Blair C., Eisen L., Elizondo-Quiroga D., Farfan-Ale J., Lozano-Fuente S., Franz A., Olson K., Atkinson P.W. (2010). Novel strategies to control *Aedes aegypti* and dengue. Vector Biology, Ecology and Control.

[5] Gubler D.J. (2002). The global emergence/resurgence of arboviral diseases as public health problems. Arch. Med. Res..

[6] Weaver S.C. (2013). Urbanization and geographic expansion of zoonotic arboviral diseases: Mechanisms and potential strategies for prevention. Trends Microbiol..

[7] Weaver S.C. (2014). Arrival of chikungunya virus in the new world: Prospects for spread and impact on public health. PLoS Negl. Trop. Dis..

[8] Weaver S.C., Reisen W.K. (2010). Present and future arboviral threats. Antivir. Res..

[9] Bisset J.A., Marin R., Rodriguez M.M., Severson D.W., Ricardo Y., French L., Diaz M., Perez O. (2013). Insecticide resistance in two *Aedes aegypti* (*Diptera*: *Culicidae*) strains from Costa Rica. J. Med. Entomol..

[10] Saavedra-Rodriguez K., Suarez A.F., Salas I.F., Strode C., Ranson H., Hemingway J., Black W.C. (2012). IV Transcription of detoxification genes after permethrin selection in the mosquito *Aedes aegypti*. Insect Mol. Biol..

[11] Villar L., Dayan G.H., Arredondo-Garcia J.L., Rivera D.M., Cunha R., Deseda C., Reynales H., Costa M.S., Morales-Ramirez J.O., Carrasquilla G. (2015). Efficacy of a tetravalent dengue vaccine in children in Latin America. N. Engl. J. Med..

[12] Alphey L., Andreasen M. (2002). Dominant lethality and insect population control. Mol. Biochem. Parasitol..

[13] Fu G., Lees R.S., Nimmo D., Aw D., Jin L., Gray P., Berendonk T.U., White-Cooper H., Scaife S., Kim Phuc H. (2010). Female-specific flightless phenotype for mosquito control. Proc. Natl. Acad. Sci. USA.

[14] Franz A.W., Sanchez-Vargas I., Raban R.R., Black W.C., James A.A., Olson K.E. (2014). Fitness impact and stability of a transgene conferring resistance to dengue-2 virus following introgression into a genetically diverse *Aedes aegypti* strain. PLoS Negl. Trop. Dis..

[15] Hardy J.L., Houk E.J., Kramer L.D., Reeves W.C. (1983). Intrinsic factors affecting vector competence of mosquitoes for arboviruses. Annu. Rev. Entomol..

[16] Romoser W.S., Beaty B.J., Marquardt W.C. (1996). The vector alimentary system. The Biology of Disease Vectors.

[17] Okuda K., de Souza Caroci A., Ribolla P.E., de Bianchi A.G., Bijovsky A.T. (2002). Functional morphology of adult female *Culex quinquefasciatus* midgut during blood digestion. Tissue Cell.

[18] Hecker H. (1977). Structure and function of midgut epithelial cells in culicidae mosquitoes (Insecta, Diptera). Cell Tissue Res..

[19] Romoser W.S., Wasieloski L.P., Pushko P., Kondic J.P., Lerdthusnee K., Neira M., Ludwig G. (2004). Evidence for arbovirus dissemination conduits from the mosquito (*Diptera*: *Culicidae*) Midgut. J. Med. Entomol..

[20] Houk E.J. (1977). Midgut ultrastructure of *Culex tarsalis* (*Diptera*: *Culcidae*) before and after a bloodmeal. Tissue Cell.

[21] Snodgrass R.E. (1959). The alimentary canal. The Anatomical Life of the Mosquito.

[22] Perrone J.B., Spielman A. (1988). Time and site of assembly of the peritrophic membrane of the mosquito *Aedes aegypti*. Cell Tissue Res..

[23] Kato N., Dasgupta R., Smartt C.T., Christensen B.M. (2002). Glucosamine:fructose-6-phosphate aminotransferase: Gene characterization, chitin biosynthesis and peritrophic matrix formation in *Aedes aegypti*. Insect Mol. Biol..

[24] Zhang M., Zheng X., Wu Y., Gan M., He A., Li Z., Liu J., Zhan X. (2010). Quantitative analysis of replication and tropisms of Dengue virus type 2 in *Aedes albopictus*. Am. J. Trop. Med. Hyg..

[25] Mourya D.T., Mishra A.C. (2000). Antigen distribution pattern of Japanese encephalitis virus in *Culex tritaeniorhynchus*, *C. vishnui* & *C. pseudovishnui*. Indian J. Med. Res..

[26] Murphy F.A., Whitfield S.G., Sudia W.D., Chamberlain R.W., Maramorosch K., Shope R.E. (1975). Interactions of vector with vertebrate pathogenic viruses. Invertebrate Immunity.

[27] Whitfield S.G., Murphy F.A., Sudia W.D. (1973). St. Louis encephalitis virus: An ultrastructural study of infection in a mosquito vector. Virology.

[28] Smith D.R., Adams A.P., Kenney J.L., Wang E., Weaver S.C. (2008). Venezuelan equine encephalitis virus in the mosquito vector *Aedes taeniorhynchus*: Infection initiated by a small number of susceptible epithelial cells and a population bottleneck. Virology.

[29] Scholle F., Girard Y.A., Zhao Q., Higgs S., Mason P.W. (2004). Trans-Packaged West Nile virus-like particles: Infectious properties *in vitro* and in infected mosquito vectors. J. Virol..

[30] Houk E.J., Kramer L.D., Hardy J.L., Chiles R.E. (1985). Western equine encephalomyelitis virus: *In vivo* infection and morphogenesis in mosquito mesenteronal epithelial cells. Virus Res..

[31] Houk E.J., Kramer L.D., Hardy J.L., Presser S.B. (1986). An interspecific mosquito model for the mesenteronal infection barrier to western equine encephalomyelitis virus (*Culex tarsalis* and *Culex pipiens*). Am. J. Trop. Med. Hyg..

[32] Girard Y.A., Popov V., Wen J., Han V., Higgs S. (2005). Ultrastructural study of West Nile virus pathogenesis in *Culex pipiens quinquefasciatus* (Diptera: Culicidae). J. Med. Entomol..

[33] Weaver S.C., Scott T.W., Lorenz L.H., Lerdthusnee K., Romoser W.S. (1988). Togavirus-associated pathologic changes in the midgut of a natural mosquito vector. J. Virol..

[34] Girard Y.A., Klingler K.A., Higgs S. (2004). West Nile virus dissemination and tissue tropisms in orally infected *Culex pipiens quinquefasciatus*. Vector Borne Zoonotic Dis..

[35] Parikh G.R., Oliver J.D., Bartholomay L.C. (2009). A haemocyte tropism for an arbovirus. J. Gen. Virol..

[36] Weaver S.C. (1986). Electron microscopic analysis of infection patterns for Venezuelan equine encephalomyelitis virus in the vector mosquito, *Culex* (Melanoconion) *taeniopus*. Am. J. Trop. Med. Hyg..

[37] Wang Z., Lu A., Li X., Shao Q., Beerntsen B.T., Liu C., Ma Y., Huang Y., Zhu H., Ling E. (2011). A systematic study on hemocyte identification and plasma prophenoloxidase from *Culex pipiens quinquefasciatus* at different developmental stages. Exp. Parasitol..

[38] Clements A.N. (1996). Adult salivary glands and their secretions. The Biology of Mosquitoes.

[39] Phattanawiboon B., Jariyapan N., Roytrakul S., Paemanee A., Sor-suwan S., Intakhan N., Chanmol W., Siriyasatien P., Saeung A., Choochote W. (2014). Morphological and protein analyses of adult female salivary glands of A*nopheles barbirostris* species A1 (*Diptera*: *Culicidae*). Trop. Biomed..

[40] Bowers D.F., Abell B.A., Brown D.T. (1995). Replication and tissue tropism of the alphavirus Sindbis in the mosquito *Aedes albopictus*. Virology.

[41] Salazar M.I., Richardson J.H., Sanchez-Vargas I., Olson K.E., Beaty B.J. (2007). Dengue virus type 2: Replication and tropisms in orally infected *Aedes aegypt*i mosquitoes. BMC Microbiol..

[42] Raquin V., Wannagat M., Zouache K., Legras-Lachuer C., Moro C.V., Mavingui P. (2012). Detection of dengue group viruses by fluorescence *in situ* hybridization. Parasit. Vectors.

[43] Janzen H.G., Rhodes A.J., Doane F.W. (1970). Chikungunya virus in salivary glands of *Aedes aegypti* (L.): An electron microscope study. Can. J. Microbiol..

[44] Gaidamovich S.Y., Khutoretskaya N.V., Lvova A.I., Sveshnikova N.A. (1973). Immunofluorescent staining study of the salivary glands of mosquitoes infected with group A arboviruses. Intervirology.

[45] Tchankouo-Nguetcheu S., Bourguet E., Lenormand P., Rousselle J.C., Namane A., Choumet V. (2012). Infection by chikungunya virus modulates the expression of several proteins in *Aedes aegypti* salivary glands. Parasit. Vectors.

[46] Ciano K.A., Saredy J.J., Bowers D.F. (2014). Heparan sulfate proteoglycan: An arbovirus attachment factor integral to mosquito salivary gland ducts. Viruses.

[47] Girard Y.A., Schneider B.S., McBee C.E., Wen J., Han V.C., Popov V., Mason P.W., Higgs S. (2007). Salivary gland morphology and virus transmission during long-term cytopathologic West Nile virus infection in *Culex* mosquitoes. Am. J. Trop. Med. Hyg..

[48] Westaway E.G., Mackenzie J.M., Kenney M.T., Jones M.K., Khromykh A.A. (1997). Ultrastructure of Kunjin virus-infected cells: Colocalization of NS1 and NS3 with double-stranded RNA, and of NS2B with NS3, in virus-induced membrane structures. J. Virol..

[49] Mackenzie J.M., Jones M.K., Young P.R. (1996). Immunolocalization of the dengue virus nonstructural glycoprotein NS1 suggests a role in viral RNA replication. Virology.

[50] Mackenzie J.M., Khromykh A.A., Jones M.K., Westaway E.G. (1998). Subcellular localization and some biochemical properties of the flavivirus Kunjin nonstructural proteins NS2A and NS4A. Virology.

[51] Forrester N.L., Coffey L.L., Weaver S.C. (2014). Arboviral bottlenecks and challenges to maintaining diversity and fitness during mosquito transmission. Viruses.

[52] Schneider B.S., Higgs S. (2008). The enhancement of arbovirus transmission and disease by mosquito saliva is associated with modulation of the host immune response. Trans. R. Soc. Trop. Med. Hyg..

[53] Black W.C., Bennett K., Gorrochotegui-Escalante N., Barillas-Mury C.V., Fernandez-Salas I., de Lourdes Munoz M., Farfan-Ale J.A., Olson K.E., Beaty B.J. (2002). Flavivirus susceptibility in *Aedes aegypti*. Arch. Med. Res..

[54] Khoo C.C.H., Piper J., Sanchez-Vargas I., Olson K.E., Franz A.W.E. (2010). The RNA interference pathway affects midgut infection- and escape barriers for Sindbis virus in *Aedes aegypti*. BMC Microbiol..

[55] Kramer L.D., Hardy J.L., Presser S.B., Houk E.J. (1981). Dissemination barriers for western equine encephalomyelitis virus in *Culex tarsalis* infected after ingestion of low viral doses. Am. J. Trop. Med. Hyg..

[56] Forrester N.L., Guerbois M., Seymour R.L., Spratt H., Weaver S.C. (2012). Vector-borne transmission imposes a severe bottleneck on an RNA virus population. PLoS Pathog..

[57] Houk E.J., Arcus Y.M., Hardy J.L., Kramer L.D. (1990). Binding of western equine encephalomyelitis virus to brush border fragments isolated from mesenteronal epithelial cells of mosquitoes. Virus Res..

[58] Woodring J., Chandler L.J., Oray C.T., McGaw M.M., Blair C.D., Beaty B.J. (1998). Short report: Diapause, transovarial transmission, and filial infection rates in geographic strains of La Crosse virus-infected *Aedes triseriatus*. Am. J. Trop. Med. Hyg..

[59] Smith D.R., Arrigo N.C., Leal G., Muehlberger L.E., Weaver S.C. (2007). Infection and dissemination of Venezuelan equine encephalitis virus in the epidemic mosquito vector, *Aedes taeniorhynchus*. Am. J. Trop. Med. Hyg..

[60] Franz A.W.E., Sanchez-Vargas I., Adelman Z.N., Blair C.D., Beaty B.J., James A.A., Olson K.E. (2006). Engineering RNA interference-based resistance to dengue virus type 2 in genetically modified *Aedes aegypti*. Proc. Natl. Acad. Sci. USA.

[61] Houk E.J., Obie F., Hardy J.L. (1979). Peritrophic membrane formation and the midgut barrier to arboviral infection in the mosquito, *Culex tarsalis* Coquillett (*Insecta*, *Diptera*). Acta Trop..

[62] Kato N., Mueller C.R., Fuchs J.F., McElroy K., Wessely V., Higgs S., Christensen B.M. (2008). Evaluation of the function of a type I peritrophic matrix as a physical barrier for midgut epithelium invasion by mosquito-borne pathogens in *Aedes aegypti*. Vector Borne Zoonotic Dis..

[63] Brown D.T., Hernandez R. (2012). Infection of cells by alphaviruses. Adv. Exp. Med. Biol..

[64] Mrkic B., Kempf C. (1996). The fragmentation of incoming Semliki Forest virus nucleocapsids in mosquito (*Aedes albopictus*) cells might be coupled to virion uncoating. Arch. Virol..

[65] Pletnev S.V., Zhang W., Mukhopadhyay S., Fisher B.R., Hernandez R., Brown D.T., Baker T.S., Rossmann M.G., Kuhn R.J. (2001). Locations of carbohydrate sites on alphavirus glycoproteins show that E1 forms an icosahedral scaffold. Cell.

[66] Myles K.M., Pierro D.J., Olson K.E. (2003). Deletions in the putative cell receptor-binding domain of Sindbis virus strain MRE16 E2 glycoprotein reduce midgut infectivity in *Aedes aegypti*. J. Virol..

[67] Pierro D.J., Myles K.M., Foy B.D., Beaty B.J., Olson K.E. (2003). Development of an orally infectious Sindbis virus transducing system that efficiently disseminates and expresses green fluorescent protein in *Aedes aegypti*. Insect Mol. Biol..

[68] Strauss J.H., Wang K.S., Schmaljohn A.L., Kuhn R.J., Strauss E.G. (1994). Host-cell receptors for Sindbis virus. Arch. Virol. Suppl..

[69] Ludwig G.V., Kondig J.P., Smith J.F. (1996). A putative receptor for Venezuelan equine encephalitis virus from mosquito cells. J. Virol..

[70] Mourya D.T., Ranadive S.N., Gokhale M.D., Barde P.V., Padbidri V.S., Banerjee K. (1998). Putative chikungunya virus-specific receptor proteins on the midgut brush border membrane of *Aedes aegypti* mosquito. Indian J. Med. Res..

[71] Klimstra W.B., Nangle E.M., Smith M.S., Yurochko A.D., Ryman K.D. (2003). DC-SIGN and l-SIGN can act as attachment receptors for alphaviruses and distinguish between mosquito cell- and mammalian cell-derived viruses. J. Virol..

[72] Liu Y., Zhang F., Liu J., Xiao X., Zhang S., Qin C., Xiang Y., Wang P., Cheng G. (2014). Transmission-blocking antibodies against mosquito C-type lectins for dengue prevention. PLoS Pathog..

[73] Rose P.P., Hanna S.L., Spiridigliozzi A., Wannissorn N., Beiting D.P., Ross S.R., Hardy R.W., Bambina S.A., Heise M.T., Cherry S. (2011). Natural resistance-associated macrophage protein is a cellular receptor for sindbis virus in both insect and mammalian hosts. Cell Host Microbe.

[74] Gubler D.J., Nalim S., Tan R., Saipan H., Sulianti Saroso J. (1979). Variation in susceptibility to oral infection with dengue viruses among geographic strains of *Aedes aegypti*. Am. J. Trop. Med. Hyg..

[75] Bosio C.F., Beaty B.J., Black W.C. (1998). Quantitative genetics of vector competence for dengue-2 virus in *Aedes aegypti*. Am. J. Trop. Med. Hyg..

[76] Mercado-Curiel R.F., Black W.C., Munoz Mde L. (2008). A dengue receptor as possible genetic marker of vector competence in *Aedes aegypti*. BMC Microbiol..

[77] Smith D.R. (2012). An update on mosquito cell expressed dengue virus receptor proteins. Insect Mol. Biol..

[78] Chen Y., Maguire T., Hileman R.E., Fromm J.R., Esko J.D., Linhardt R.J., Marks R.M. (1997). Dengue virus infectivity depends on envelope protein binding to target cell heparan sulfate. Nat. Med..

[79] Chu J.J., Leong P.W., Ng M.L. (2005). Characterization of plasma membrane-associated proteins from *Aedes albopictus* mosquito (C6/36) cells that mediate West Nile virus binding and infection. Virology.

[80] Muñoz M.L., Cisneros A., Cruz J., Das P., Tovar R., Ortega A. (1998). Putative dengue virus receptors from mosquito cells. FEMS Microbiol. Lett..

[81] Mercado-Curiel R.F., Esquinca-Aviles H.A., Tovar R., Diaz-Badillo A., Camacho-Nuez M., Muñoz Mde L. (2006). The four serotypes of dengue recognize the same putative receptors in *Aedes aegypti* midgut and *Ae. albopictus* cells. BMC Microbiol..

[82] Sakoonwatanyoo P., Boonsanay V., Smith D.R. (2006). Growth and production of the dengue virus in C6/36 cells and identification of a laminin-binding protein as a candidate serotype 3 and 4 receptor protein. Intervirology.

[83] Salas-Benito J.S., del Angel R.M. (1997). Identification of two surface proteins from C6/36 cells that bind dengue type 4 virus. J. Virol..

[84] Salas-Benito J., Reyes-Del Valle J., Salas-Benito M., Ceballos-Olvera I., Mosso C., del Angel R.M. (2007). Evidence that the 45-kD glycoprotein, part of a putative dengue virus receptor complex in the mosquito cell line C6/36, is a heat-shock related protein. Am. J. Trop. Med. Hyg..

[85] Kuadkitkan A., Wikan N., Fongsaran C., Smith D.R. (2010). Identification and characterization of prohibitin as a receptor protein mediating DENV-2 entry into insect cells. Virology.

[86] Muñoz Mde L., Limon-Camacho G., Tovar R., Diaz-Badillo A., Mendoza-Hernandez G., Black W.C. (2013). Proteomic identification of dengue virus binding proteins in *Aedes aegypti* mosquitoes and *Aedes albopictus* cells. Biomed. Res. Int..

[87] Bennett K.E., Flick D., Fleming K.H., Jochim R., Beaty B.J., Black W.C.T. (2005). Quantitative trait loci that control dengue-2 virus dissemination in the mosquito *Aedes aegypti*. Genetics.

[88] Turell M.J., Gargan T.P., Bailey C.L. (1984). Replication and dissemination of Rift Valley fever virus in *Culex pipiens*. Am. J. Trop. Med. Hyg..

[89] Weaver S.C., Scherer W.F., Cupp E.W., Castello D.A. (1984). Barriers to dissemination of Venezuelan encephalitis viruses in the Middle American enzootic vector mosquito, *Culex* (Melanoconion) taeniopus. Am. J. Trop. Med. Hyg..

[90] Yurchenco P.D., O’Rear J.J. (1994). Basal lamina assembly. Curr. Opin. Cell. Biol..

[91] Grimstad P.R., Walker E.D. (1991). Aedes triseriatus (Diptera: Culicidae) and La Crosse virus. IV. Nutritional deprivation of larvae affects the adult barriers to infection and transmission. J. Med. Entomol..

[92] Thomas R.E., Wu W.K., Verleye D., Rai K.S. (1993). Midgut basal lamina thickness and dengue-1 virus dissemination rates in laboratory strains of *Aedes albopictus* (*Diptera*: *Culicidae*). J. Med. Entomol..

[93] Chandler L.J., Blair C.D., Beaty B.J. (1998). La Crosse virus infection of *Aedes triseriatus* (Diptera: Culicidae) ovaries before dissemination of virus from the midgut. J. Med. Entomol..

[94] Engelhard E.K., Kam-Morgan L.N.W., Washburn J.O., Volkman L.E. (1994). The insect tracheal system: A conduit for the systemic spread of *Autographa californica* M nuclear polyhedrosis virus. Proc. Natl. Acad. Sci. USA.

[95] Kirkpatrick B.A., Washburn J.O., Engelhard E.K., Volkman L.E. (1994). Primary infection of insect tracheae by *Autographa californica* M nuclear polyhedrosis virus. Virology.

[96] Rahman M.M., Gopinathan K.P. (2004). Systemic and *in vitro* infection process of *Bombyx mori* nucleopolyhedrovirus. Virus Res..

[97] Romoser W.S., Turell M.J., Lerdthusnee K., Neira M., Dohm D., Luldwig G., Wasieloski L. (2005). Pathogenesis of Rift Valley fever virus in mosquitoes-tracheal conduits and the basal lamina as an extra-cellular barrier. Arch. Virol. Suppl..

[98] Oviedo M.V., Romoser W.S., James C.B., Mahmood F., Reisen W.K. (2011). Infection dynamics of western equine encephalomyelitis virus (*Togaviridae*: *Alphavirus*) in four strains of *Culex tarsalis* (*Diptera*: *Culicidae*): An immunocytochemical study. Res. Rep. Trop. Med..

[99] Passarelli A.L. (2011). Barriers to success: How baculoviruses establish systemic infections. Virology.

[100] Myles K.M., Pierro D.J., Olson K.E. (2004). Comparison of the transmission potential of two genetically distinct Sindbis viruses after oral infection of *Aedes aegypti* (*Diptera*: *Culicidae*). J. Med. Entomol..

[101] Granados R.R., Lawler K.A. (1981). *In vivo* pathway of *Autographa californica* baculovirus invasion and infection. Virology.

[102] Means J.C., Passarelli A.L. (2010). Viral fibroblast growth factor, matrix metalloproteases, and caspases are associated with enhancing infection by baculoviruses. Proc. Natl. Acad. Sci. USA.

[103] Romoser W.S., Faran M.E., Bailey C.L., Lerdthusnee K. (1992). An immunocytochemical study of the distribution of Rift Valley fever virus in the mosquito *Culex pipiens*. Am. J. Trop. Med. Hyg..

[104] Lerdthusnee K., Romoser W.S., Faran M.E., Dohm D.J. (1995). Rift Valley fever virus in the cardia of *Culex pipiens*: An immunocytochemical and ultrastructural study. Am. J. Trop. Med. Hyg..

[105] Brault A.C., Foy B.D., Myles K.M., Kelly C.L., Higgs S., Weaver S.C., Olson K.E., Miller B.R., Powers A.M. (2004). Infection patterns of o’nyong nyong virus in the malaria-transmitting mosquito, *Anopheles gambiae*. Insect Mol. Biol..

[106] Linthicum K.J., Platt K., Myint K.S., Lerdthusnee K., Innis B.L., Vaughn D.W. (1996). Dengue 3 virus distribution in the mosquito *Aedes aegypti*: An immunocytochemical study. Med. Vet. Entomol..

[107] Foy B.D., Myles K.M., Pierro D.J., Sanchez-Vargas I., Uhlirova M., Jindra M., Beaty B.J., Olson K.E. (2004). Development of a new Sindbis virus transducing system and its characterization in three Culicine mosquitoes and two lepidopteran species. Insect Mol. Biol..

[108] Paulson S.L., Grimstad P.R., Craig G.B. (1989). Midgut and salivary gland barriers to La Crosse virus dissemination in mosquitoes of the *Aedes triseriatus* group. Med. Vet. Entomol..

[109] Scott T.W., Lorenz L.H., Weaver S.C. (1990). Susceptibility of *Aedes albopictus* to infection with eastern equine encephalomyelitis virus. J. Am. Mosq. Control Assoc..

[110] Turell M.J., Mores C.N., Dohm D.J., Komilov N., Paragas J., Lee J.S., Shermuhemedova D., Endy T.P., Kodirov A., Khodjaev S. (2006). Laboratory transmission of Japanese encephalitis and West Nile viruses by molestus form of *Culex pipiens* (*Diptera*: *Culicidae*) collected in Uzbekistan in 2004. J. Med. Entomol..

[111] Dourado L.A., Ribeiro L.F., Brancalhao R.M., Tavares J., Borges A.R., Fernandez M.A. (2011). Silkworm salivary glands are not susceptible to *Bombyx mori* nuclear polyhedrosis virus. Genet. Mol. Res..

[112] Barreau C., Conrad J., Fischer E., Lujan H.D., Vernick K.D. (1999). Identification of surface molecules on salivary glands of the mosquito, *Aedes aegypti*, by a panel of monoclonal antibodies. Insect Biochem. Mol. Biol..

[113] Juhn J., Naeem-Ullah U., Maciel Guedes B.A., Majid A., Coleman J., Paolucci Pimenta P.F., Akram W., James A.A., Marinotti O. (2011). Spatial mapping of gene expression in the salivary glands of the dengue vector mosquito, *Aedes aegypti*. Parasit. Vectors.

[114] Rossignol P.A., Spielman A.J. (1982). Fluid transport across the ducts of the salivary glands of a mosquito. Insect Physiol..

[115] Beaty B.J., Holterman M., Tabachnick W., Shope R.E., Rozhon E.J., Bishop D.H. (1981). Molecular basis of bunyavirus transmission by mosquitoes: Role of the middle-sized RNA segment. Science.

[116] Grimstad P.R., Paulson S.L., Craig G.B. (1985). Vector competence of *Aedes hendersoni* (*Diptera*: *Culicidae*) for La Crosse virus and evidence of a salivary-gland escape barrier. J. Med. Entomol..

[117] Jupp P.G. (1985). *Culex theileri* and Sindbis virus; salivary glands infection in relation to transmission. J. Am. Mosq. Control Assoc..

[118] Paulson S.L., Poirier S.J., Grimstad P.R., Craig G.B. (1992). Vector competence of *Aedes hendersoni* (*Diptera*: *Culicidae*) for La Crosse virus: Lack of impaired function in virus-infected salivary glands and enhanced virus transmission by sporozoite-infected mosquitoes. J. Med. Entomol..

[119] Turell M.J., Britch S.C., Aldridge R.L., Kline D.L., Boohene C., Linthicum K.J. (2013). Potential for mosquitoes (*Diptera*: *Culicidae*) from Florida to transmit Rift Valley fever virus. J. Med. Entomol..

[120] Campbell C.L., Keene K.M., Brakney D.E., Olson K.E., Blair C.D., Wilusz J., Foy B.D. (2008). *Aedes aegypti* uses RNA interference in defense against Sindbis virus infection. BMC Microbiol..

[121] Khoo C.C., Doty J.B., Heersink M.S., Olson K.E., Franz A.W. (2013). Transgene-mediated suppression of the RNA interference pathway in *Aedes aegypti* interferes with gene silencing and enhances Sindbis virus and dengue virus type 2 replication. Insect Mol. Biol..

[122] Sanchez-Vargas I., Scott J.C., Poole-Smith B.K., Franz A.W., Barbosa-Solomieu V., Wilusz J., Olson K.E., Blair C.D. (2009). Dengue virus type 2 infections of *Aedes aegypti* are modulated by the mosquito’s RNA interference pathway. PLoS Pathog..

[123] Kelly E.M., Moon D.C., Bowers D.F. (2012). Apoptosis in mosquito salivary glands: Sindbis virus-associated and tissue homeostasis. J. Gen. Virol..

[124] Vaidyanathan R., Scott T.W. (2006). Apoptosis in mosquito midgut epithelia associated with West Nile virus infection. Apoptosis.

[125] Wang H., Gort T., Boyle D.L., Clem R.J. (2012). Effects of manipulating apoptosis on Sindbis virus infection of *Aedes aegypti* mosquitoes. J. Virol..

[126] Liu Q., Clem R.J. (2011). Defining the core apoptosis pathway in the mosquito disease vector *Aedes aegypti* the roles of *iap1*, *ark*, *dronc*, and effector caspases. Apoptosis.

[127] O’Neill K., Olson B.J., Huang N., Unis D., Clem R.J. (2015). Rapid selection against arbovirus-induced apoptosis during infection of a mosquito vector. Proc. Natl. Acad. Sci. USA.

[128] Pierro D.J., Powers E.L., Olson K.E. (2007). Genetic determinants of Sindbis virus strain TR339 affecting midgut infection in the mosquito *Aedes aegypti*. J. Gen. Virol..

[129] Pierro D.J., Powers E.L., Olson K.E. (2008). Genetic determinants of Sindbis virus mosquito infection are associated with a highly conserved alphavirus and flavivirus envelope sequence. J. Virol..

[130] Khoo C.C., Doty J.B., Held N.L., Olson K.E., Franz A.W. (2013). Isolation of midgut escape mutants of two American genotype dengue 2 viruses from *Aedes aegypti*. Virol. J..

